# Perceived Usefulness of Telerehabilitation of Musculoskeletal Disorders: A Belgium–France Pilot Study during Second Wave of COVID-19 Pandemic

**DOI:** 10.3390/healthcare9111605

**Published:** 2021-11-22

**Authors:** Frédéric Dierick, Amélie Pierre, Loredana Profeta, Frédéric Telliez, Fabien Buisseret

**Affiliations:** 1 CeREF Technique, Chaussée de Binche 159, 7000 Mons, Belgium; frederic.dierick@gmail.com; 2 Faculté des Sciences de la Motricité, Université Catholique de Louvain, Place Pierre de Coubertin 2, 1348 Louvain-la-Neuve, Belgium; 3 Laboratoire d’Analyse du Mouvement et de la Posture (LAMP), Centre National de Rééducation Fonctionnelle et de Rédaptation—Rehazenter, Rue André Vésale 1, 2674 Luxembourg, Luxembourg; 4 Centre FoRS, Département Social Namur, Domaine de l’information et de la Communication et Domaine des Sciences Politiques et Sociales, Haute École Namur-Liège-Luxembourg—HENALLUX, Rue de l’Arsenal 10, 5000 Namur, Belgium; amelie.pierre@henallux.be; 5 Institut Transitions, UNamur, Rue de Bruxelles 61, 5000 Namur, Belgium; 6 Forme and Fonctionnement Humain Lab, Department of Physical Therapy, Haute Ecole Louvain en Hainautrue, Trieu Kaisin 136, 6061 Montignies sur Sambre, Belgium; profeta.loredana@hotmail.com; 7 Institut d’Ingénierie de la Santé-UFR de Médecine, Université de Picardie Jules Verne, Rue des Louvels, 80036 Amiens, France; frederic.telliez@u-picardie.fr; 8 Centre Universitaire de Recherche en Santé-Laboratoire Péritox (UMR_01), Chemin du Thil, Présidence, Université de Picardie Jules Verne, 80025 Amiens, France; 9 Service de Physique Nucléaire et Subnucléaire, UMONS Research Institute for Complex Systems, Université de Mons, 20 Place du Parc, 7000 Mons, Belgium

**Keywords:** telerehabilitation, musculoskeletal disease, survey, e-health, acceptability

## Abstract

Background: COVID-19 has affected the practice of physiotherapy, and telerehabilitation (TR) may be seen as an alternative model of care if it is accepted by patients and physiotherapists. This study investigates the perceived usefulness of TR and the intention to use it among physiotherapists and patients from Belgium and France concerned with musculoskeletal disorders (MSDs) during the pandemic period. Methods: An online questionnaire based on the technology-acceptance model was designed. Sociodemographic data were collected and Likert scales were proposed to assess perceived ease-of-use, perceived usefulness and intention to use TR. Data were collected between 17 January and 17 March 2021; 68 patients and 107 physiotherapists answered. Results: In total, 88% of patients and 76% physiotherapists had not used TR at the time they answered. Only 12% of patients and 1% of physiotherapists are willing to use TR, and 50% of physiotherapists think they will never use TR compared to 25% of patients. A total of 98% of participants agreed that they had a good mastery of the technological tools requested. Conclusions: Physiotherapists are more reluctant to use TR than patients, regardless of convincing EBM results. This is related to their own representation of proper MSD management, which must include the use of hands-on techniques.

## 1. Introduction

The 2019 Global Burden of Disease (GBD) data indicate that 2.41 billion people have conditions that would benefit from rehabilitation, and the disease area that contributes by far the most to prevalence is musculoskeletal disorders (MSDs), with 1.71 billion people [1]; these encompass low back pain at 568 million people, fractures at 436 million, osteoarthritis at 343 million, other injuries at 305 million, neck pain at 223 million, amputations at 176 million and rheumatoid arthritis at 13 million [1]. The rehabilitation of MSDs is, therefore, an important component of healthcare, and in particular, physiotherapy. COVID-19 has strongly affected the delivery of healthcare in physiotherapy. In response, telerehabilitation (TR) has emerged as an alternative care model [2].

By definition, TR is the ensemble of rehabilitation techniques relying on information and communication technologies (ICTs), regardless of the patient’s and therapist’s geographic location [3]. TR has experienced a renewed interest because of the COVID-19 pandemic, since it is a process that facilitates the continuity of care for patients suffering from MSDs [4]. However, TR remains different from traditional rehabilitation because hands-on therapeutic approaches during treatment, meeting other patients in the waiting room, noises or smells related to the treatment room (which may impact the effectiveness of the treatment), and clinical measurement tools are mostly absent during remote consultations [4]. Although the most basic modality used for TR is the telephone interview, video-based TR is necessary in the field of MSDs to maintain patients’ adherence to treatment and motivation [5]. Video-based TR demands access to the internet. In 2019, in Belgium, the rate of households with access to the internet was 90%, while 7% of people had never used it [6]. In France, in 2020, 89% of the population had access to the internet [7]. In the European Union, 90% of the population has access to the internet [8]. In the following, we focus on Belgium and France, as the present study is part of the France–Wallonie–Vlaanderen Interreg project entitled NOMADe (NeurOMuskuloskeletAl Disorders—e-learning ecosystem, http://nomadeproject.eu/ (accessed on 1 November 2021)). Belgium and France are the two countries targeted by the France–Wallonie–Vlaanderen Interreg program.

A systematic review including 11 studies shows that physiotherapy assessment is technically feasible by TR for various MSDs with good-to-excellent concurrent validity and reliability for range of motion, muscle strength, gait, and balance [9]. However, TR is not a flawless modality since lumbar spine posture assessment, special orthopedic and neurodynamic tests, and scar assessment showed low-to-moderate concurrent validity [9]. TR also gives positive results in MSDs treatment [10,11,12]. The peculiarities of TR are such that it is not obvious that it can be easily accepted by physiotherapists and/or patients. First, physiotherapy is a profession where, in a commonly accepted image, physical contact with the patient is omnipresent through “hands-on” treatment. Second, the use of TR demands minimal skills in mastering ICTs. The most widely used model to predict individual adoption and use of new ICTs, including telemedicine, is the technology acceptance model (TAM) [13,14]. The model assumes that individuals’ behavioral intention to use an ICT is determined by two beliefs: perceived usefulness and perceived ease of use. Perceived usefulness is defined as the extent to which an individual believes that using an ICT will enhance his or her job performance, and perceived ease of use as the degree to which an individual believes that using an ICT will be free of effort. Several upgrades to this model are available. Here, we used the TAM 3 model [15], which combines TAM 2 [16] and the model of the determinants of perceived ease of use [17]. In this framework, general determinants of perceived usefulness are the following: subjective norm, image, job relevance, output quality, and result demonstrability. The first three determinants fall into the category of social influence, and the remaining are system characteristics. Such a model is a priori relevant for our purpose since these social determinants may be linked to the physiotherapist’s professional identity and one of its main dimensions: the face-to-face, physical and tactile nature of therapeutic practice [18]. Indeed, perceived usefulness is linked to this fundamental element of the physiotherapist’s expertise: presence and contact. Note that a TAM-based approach has already been used to assess physiotherapists’ intention to use a mobile movement monitoring platform in the field of neurorehabilitation, and a moderate-to-high intention was found [19]. Moreover, in [20], TAM has proved to be appropriate in exploring physiotherapists’ attitudes toward incorporating mobile or wearable technology into their practice.

How did patients and physiotherapists from Belgium and France perceive the usefulness of TR during the COVID-19 pandemic period? We address this question with an online questionnaire composed of items inspired by TAM 3 [15]. As a side question, we also examine whether this parameter influences the intention to use TR. Indeed, it seems logical that younger generations are more likely to endorse and/or support the use of TR, given their familiarity with these techniques. To our knowledge, this is the first time that the attitudes of both patients and physiotherapists towards TR have been investigated in the field of MSDs.

## 2. Materials and Methods

### 2.1. Questionnaire

This study investigates the perceived usefulness of TR among physiotherapists and patients concerned with MSDs by means of an online questionnaire. The questionnaire was designed on Forms (Microsoft Corporation, Redmond, WA, USA) and was composed of several parts that will be detailed below. The French version of the questionnaire was submitted to an independent ethics committee (see Institutional Review Board Statement) before the start of the study. Subsequently, the questionnaire was sent to a Belgian translation agency to be translated into Dutch so that it could be distributed in Flanders. The questionnaire was distributed between 17 January and 17 March 2021 (weeks 2 to 11). This period was referred to as the second wave of COVID-19, i.e., the second peak of daily COVID-19 infections since the pandemic’s beginning in Belgium and France. Some participants were contacted by email, through a Facebook post and on LinkedIn. A poster with a QR code linking to the questionnaire was also distributed in France and Wallonia by e-mail and on social networks.

Before starting to fill in the questionnaire, all the participants (patients and physiotherapists) had to “sign” an informed consent by ticking a specific box. By giving their consent, participants confirmed that they understood: (1) the information about the study; (2) the confidentiality of the data collected for scientific research purposes; (3) that they could contact the research team if they had any further questions. Participants were also asked whether they were responding as a physiotherapist or a patient, and whether they were concerned or not with MSDs. A patient concerned with MSDs is defined, at the time of the study, as “suffering from at least one MSD” and a physiotherapist concerned with MSDs is defined as “managing patients suffering from MSDs”. Only the responses of participants concerned with MSD-related responses were collected and analyzed. Participants giving incoherent information, e.g., incorrect zip code regarding the country, were excluded. A contradiction between a physiotherapist’s answers to the number of patients managed with TR and the previous use of TR also led to exclusion.

**Figure 1:**
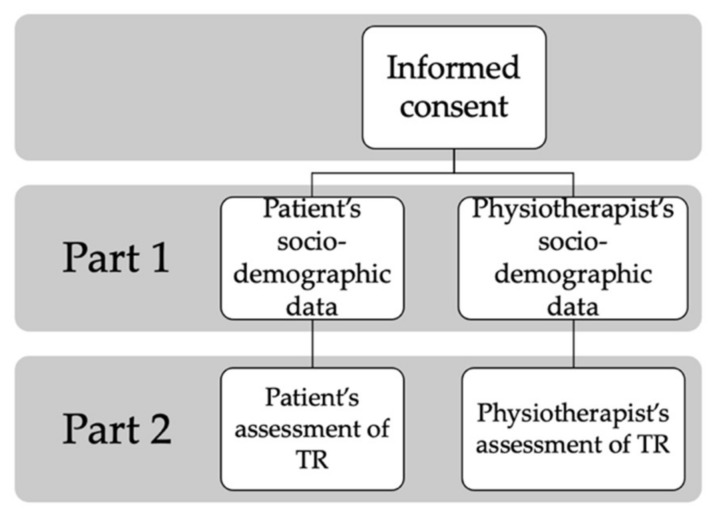
Schematic representation of our questionnaire’s structure.

Then, the two main parts of the questionnaire followed. Their structure is illustrated in Figure 1. The first part of the questionnaire was designed to record the sociodemographic data of the participants: age, the highest diploma obtained (from secondary school to doctorate), place of residence or occupation, zip code, the existence of access to the Internet, rating of their Internet access (accessibility and quality), the location of the MSD for the patients or the number of different MSDs patients treated per week during the last 6 months, face-to-face and with TR for the physiotherapists. The second part of the questionnaire focused on several aspects related to TR. First, on the previous use of TR:

- Whether the participant had previously used TR for the management of MSDs and/or other disorders;
- For what reasons it was used: sanitary measures, lack of transport, inability to reach the site or other;
- The modalities used, i.e., phone, smartphone, computer, tablet, or other.

Second, a series of Likert scales with 5 grades (disagree, mostly disagree, agree, mostly agree, no opinion) assessed how the participant felt about different TAM-related concepts. The different items are presented in Table A1 (Appendix A). This part of the questionnaire was mainly inspired by TAM 3, which has the benefit of not assuming any crossover effects between perceived usefulness and perceived ease of use [15]. The item related to intention to use TR was also included to assess behavioral intention. The 5 grades of our Likert scales are such that they forbid a neutral answer: If the participant has an opinion, he/she must either agree or disagree. Finally, the main advantage participants felt TR had among these proposals was asked: time saving, travel saving, health safety, flexibility in planning and duration of sessions and increased autonomy in management.

Before the questionnaire was distributed, a test group of six participants (3 physiotherapists and 3 patients) was set up to obtain some advice on the items of the questionnaire and to confirm if the wording allowed the participants to understand the items properly.

### 2.2. Population

Inclusion criteria for patients were: living in Belgium or France; have been or be treated for at least one MSD (chronic or acute) within 3 years, with or without TR; over 18 years old. Exclusion criteria for patients were: absence of MSD, questionnaire not fully completed. Inclusion criteria for physiotherapists were: working in Belgium or France, being active in the management of patients suffering from MSDs, either in face-to-face or via TR. Exclusion criteria were: absence of MSD management for the physiotherapists and questionnaire not fully completed.

In the end, 132 physiotherapists who had treated patients with MSDs and 72 patients who had been treated for MSDs responded to the questionnaire. Participants who had not experienced MSDs were excluded (12 physiotherapists and 17 patients). Of the 132 physiotherapists, 25 contradicted themselves by answering the question about the number of different patients seen by TR and also answering that they had never practiced it. Finally, 107 physiotherapists were included in this study. Of the 72 patients, there was one who gave a wrong zip code and one who did not give a zip code. Two others said they had never suffered from an MSD. These 4 patients were therefore excluded, and 68 patients were left in the study.

### 2.3. Data Analysis

Data from physiotherapists and patients concerned with MSDs were collected and the percentages of answers to the different questions were computed. Cronbach’s α values for patients and physiotherapists have been computed for the answers to the items listed in Table A1 (1 = disagree, 2 = mostly disagree, 3 = agree, 4 = mostly agree, 5 = no opinion) by using R free software (version 4.1.0). This assesses the reliability (internal consistency) of our questionnaire. Various figures were then produced in the form of bar and pie charts to graphically explore the results. X^2^ tests were then performed to compare patients’ and physiotherapists’ answers, and a *t*-test was used to compare patients’ and physiotherapists’ ages. SigmaPlot software (version 11.1, Systat Software, San Jose, CA, USA) was used with a significance threshold of 0.05.

Then, the physiotherapists were divided into two groups according to the threshold of a logistic regression of their intention to use TR (0 = never, 1 = other choice) versus their age. This defines groups of “young” and “old” physiotherapists. X^2^ tests were then performed to compare young and old physiotherapists’ answers.

## 3. Results

### 3.1. Participants’ Sociodemographic Data

Descriptive data concerning patients and physiotherapists are shown in Table 1. Patients are 15 years older than physiotherapists in our study (*p* ≤ 0.001). It appears that almost all the participants declared having an adequate connection to the internet and an adequate ease to use it. A great majority of patients and physiotherapists had not used TR at the time that they answered the questionnaire.

Extra data have been recorded: we now summarize them. Physiotherapists have resorted to TR because of lockdown (91%) or temporary inability to travel (9%). They used various technologies: phone (11%), smartphone (30%), PC (41%), tablet (18%). The main advantages of TR according to physiotherapists are sanitary safety (25%) and increased autonomy in management (25%). Patients’ MSDs were located at the head and neck (12%), upper limb (16%), lumbar region (28%) and lower limb (20%). The main advantages of TR according to patients are sanitary safety (47%) and time saving (24%).

### 3.2. Patients vs. Physiotherapists

**Figure 2:**
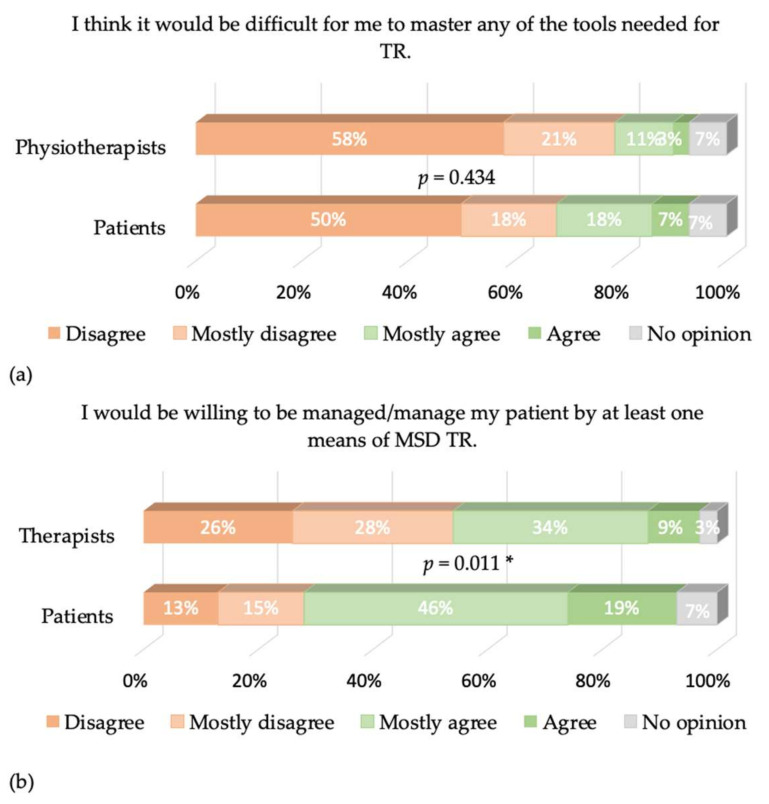
(**a**) Answers from physiotherapists and patients on the ease-of-use-related item. The *p*-value of the X^2^ test comparing the percentages is given. (**b**) Same as (**a**) for the image-related item. A * denotes a significant difference in the percentages. * means *p* < 0.05.

**Figure 3:**
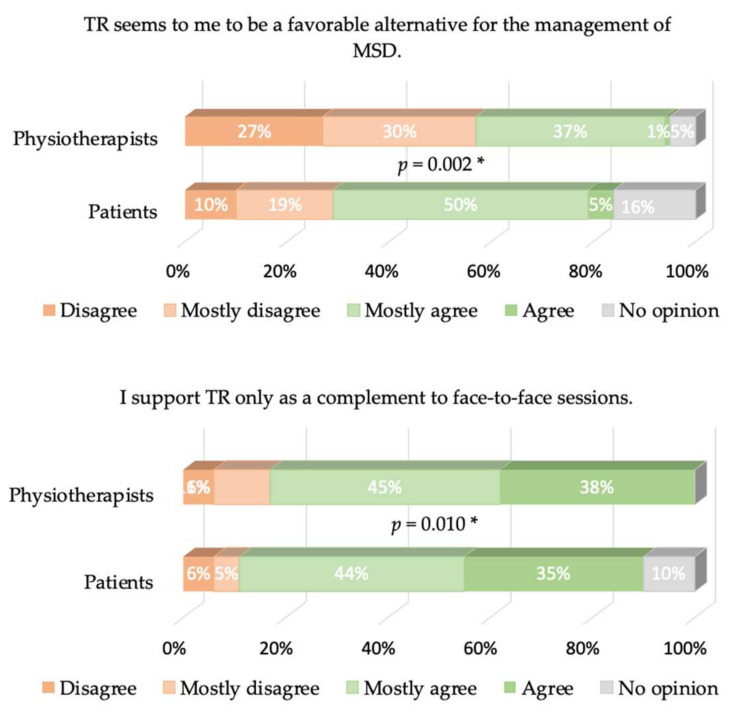
Same as Figure 2 for the job-relevance-related items. * means *p* < 0.05.

**Figure 4:**
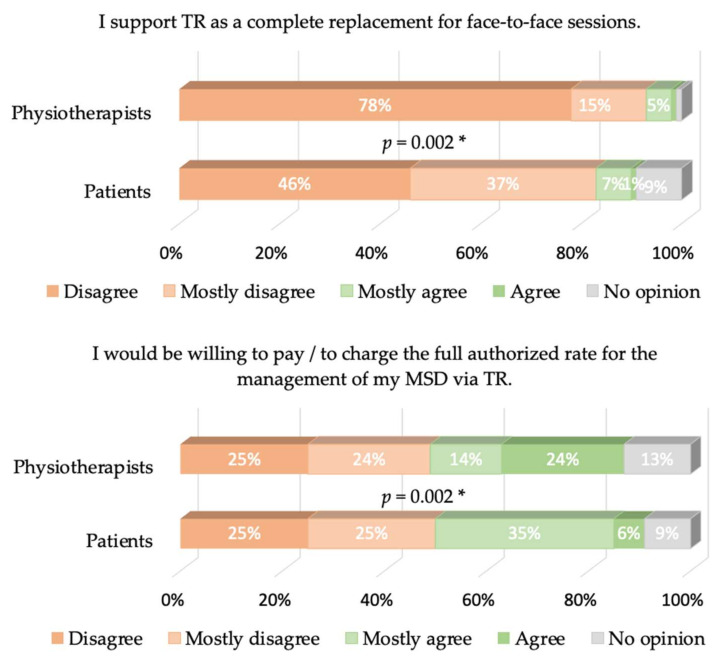
Same as Figure 2 for the output-quality-related items. Upper plot: The 1% “agree” and the 1% “no opinion” were not displayed in the physiotherapists’ answers for the sake of clarity. * means *p* < 0.05.

**Figure 5:**
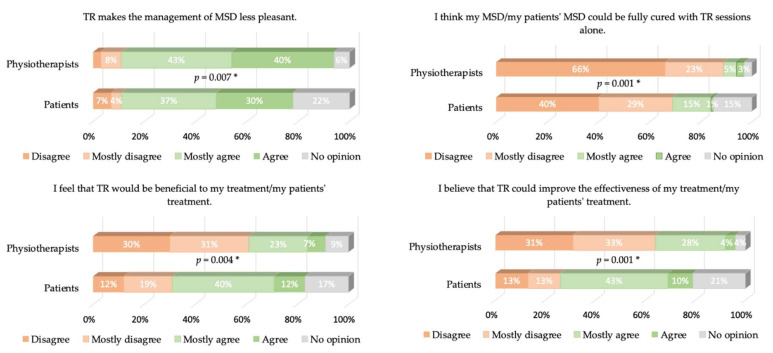
Same as Figure 2 for the result-demonstrability-related items. Upper right plot: The 3% “no opinion” in the physiotherapists’ answers were not displayed for the sake of clarity. * means *p* < 0.05.

**Figure 6:**
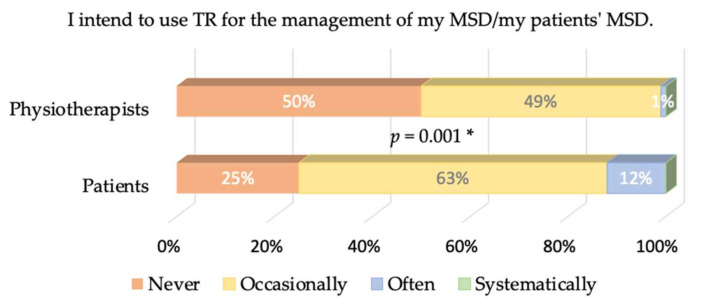
Answers from the physiotherapists and patients to the behavioral-intention-related item. The *p*-value of the X^2^ test comparing the percentages is given. A * denotes a significant difference in the percentages. * means *p* < 0.05.

Cronbach’s α values were equal to 0.77 and 0.68 for patients and physiotherapists, respectively. The answers to the different items of our questionnaire are presented in Figure 2, Figure 3, Figure 4, Figure 5 and Figure 6, where the trends and differences between both groups can be graphically appraised. It must be noted that percentages are significantly different for all items, except the one concerning the perceived ease of use of TR tools.

One extra item has been asked to patients only: “I think that understanding the exercises demanded is more difficult in TR than in face-to-face”. The answers were: 24% disagree, 29% mostly disagree, 24% mostly agree, 18% agree and 6% no opinion.

### 3.3. Young vs. Old Physiotherapists

**Figure 7:**
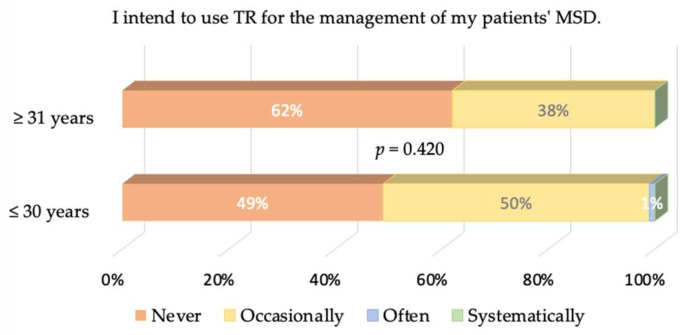
Same as Figure 6 but for physiotherapists older and younger than 30.5 years.

The logistic regression was logit = 0.9560 − 0.0313 age, leading to a threshold of $\frac{0.9560}{0.0313}=30.5$ years. We kept this value to divide our population into young (age ≤ 30 years) and old (age ≥ 31 years). We stress that the *p*-values associated with the intercept and the slope are equal to 0.1070 and 0.0747, respectively: although older therapists tend to answer that they will never use TR more often than younger ones, this trend may not be seen as significant. It follows that the answers of both groups to the different items were never significantly different, so we do not show all the plots for the sake of simplicity. Only the intention of both groups to use TR in the future is graphically displayed in Figure 7 for completeness.

## 4. Discussion

We conducted an online survey based on TAM3 to assess patients’ and physiotherapists’ perceived ease-of-use, perceived usefulness and intention to use TR in the management of MSDs. Patients’ and physiotherapists’ opinions were compared, and the influence of the physiotherapist’s age on the intention to use TR was assessed. Regarding the reliability of our questionnaire, Cronbach’s α values above or compatible with 0.7 were found, which means that the reliability can be considered satisfactory.

We first comment on our population’s features. The MSDs affecting the patients in our study appear to be representative of the distribution of MSDs in Belgium and France [22]. A great majority of physiotherapists (76%) and patients (88%) had never used TR at the time that they answered the questionnaire, despite the first lockdown induced by COVID-19 in early 2020 and the high number of deaths per million inhabitants in the targeted zones [23]. We have therefore primarily collected a priori opinions.

A key result of our study is that 50% of physiotherapists state that they would never use TR compared to 25% of patients, and 12% of patients would often use TR, while only 1% of physiotherapists believe this. Behavioral intention is negative overall and more salient in physiotherapists. This does not originate from the perceived ease of use of TR: The great majority of both physiotherapists and patients have satisfactory access to the internet and think that mastering the TR tools would not be problematic (79% of the physiotherapists and 68% of the patients think so). This is expected since the questionnaire was distributed using emails and social networks—the population reached is used to ICTs.

Perceived usefulness is, therefore, mostly responsible for the global lack of acceptance of TR. A total of 57% of physiotherapists do not think that TR is a favorable alternative for managing TR, while only 29% of the patients think so (and 16% have no opinion). As a logical consequence, 94% of the physiotherapists think TR cannot completely replace face-to-face sessions, as well as 83% of the patients. Furthermore, 83% of physiotherapists and 79% of patients think that TR may be used as a complement of face-to-face sessions; only 30% of physiotherapists think that TR may be beneficial for their patient’s treatment, while 52% of patients think it may be. Another striking observation is that only 30% of the physiotherapists believe that TR would be beneficial to their patient’s treatment, while 52% of the patients believe it. This belief is actually in contradiction with the evidence-based practice/medicine (EBP/M) literature, as discussed in the Introduction. We add that more recent references on the same topic can be found in the masterclass paper [24].

The strong rejection of TR by physiotherapists must now be discussed. An obvious question: May it be a matter of generation? In our study, young physiotherapists belong to Y or Z generations. During their studies and careers, they have always lived with social networks, and have even used several different networks simultaneously. A total of 54% of the younger physiotherapists would use TR occasionally or often, versus 46% of the older physiotherapists, but this difference is not significant. Two other hypotheses may be expressed about physiotherapists’ rejection of TR: (1) they are not aware of the literature showing the interest in TR, and (2) their own representation of professional identity, i.e. a subjective phenomenon in which people’s individual dispositions, such as attributes, beliefs, values, motives, and experiences [25], about what constitutes a “good” physiotherapy session precludes the use of TR. The authors of [26] highlight three identity logics within the profession of physiotherapist (masseur-kinésithérapeute) in France: vocational, evolutionary and expertise. These three logics are simultaneously articulated within one clinician. Expertise enables the individual to distinguish himself from other professions or colleagues. The relationship with the patient’s body is “the heart of the profession” of physiotherapists [27], i.e., a crucial part of expertise. In other words, the “body to body”, the physical proximity to the patient, is a fundamental element of their professional identity [18]. At the time of data acquisition, TR was officially reimbursed by INAMI in Wallonia and RIZIV in Flanders (Belgium), and by the National Institute for Health and Disability Insurance in France [28]. The fact that 62% of physiotherapists would not accept charging their patients despite the possibility of being reimbursed further shows how TR goes against their conception of what a “good” management of MSD is. As a consequence, the job relevance of TR is not perceived by physiotherapists in the management of MSDs, a conclusion that we share with an internet survey of health professionals working in pulmonary rehabilitation [29]. Note that more positive opinions may be observed in other fields such as neurorehabilitation [19], where hands-off techniques are more common.

Our results show that patients are significantly less opposed to TR than therapists; time saving and sanitary safety are quoted most as advantages. We think that these findings are coherent with previous studies showing that patients agree to use TR when they have no other option, but still prefer traditional consultations [30,31,32]. It was also shown in [33] that a significant reduction in pain, anxiety and depression was possible with TR when patients worked independently. Patient autonomy is thus an important positive element of TR. According to [34], TR could finally be a good alternative to treat patients living in underserved areas. Some authors also claim that TR could reduce the hospitalization rate of patients with MSDs, since they can receive their care remotely, as well as reduce their readmission and decrease their length of stay [35].

Finally, several limitations have to be mentioned. The number of participants in our study can be seen as a first obvious limitation. A second one is that participants were recruited by mail on social networks: we exclude de facto a population that is unfamiliar with these technologies. Note, however, that the excluded patients and physiotherapists should not have an opinion about TR as relevant as those familiar with ICTs. A last limitation is that we did not ask physiotherapists whether or not they were aware of the EBP/M results about TR.

## 5. Concluding Comments

In our opinion, TR of MSDs was a relevant alternative to face-to-face sessions since the first COVID-19 lockdown. However, physiotherapists and patients are against it according to our survey, with patients being slightly more optimistic about TR in complement to face-to-face sessions. In [36], a study investigating physiotherapists’ opinions on the effectiveness of TR for managing low back pain was conducted. In all, it was found that physiotherapists believe that TR can only be effective if the patient is involved in the treatment. The patient, therefore, also has a fundamental role to play in the possible implementation of TR.

In the context of prevention and limitation of physiological and functional alterations in the elderly, the superior impact of home training with distance supervision compared to semi- or unsupervised training has been shown in [37]. A simple example of TR implementation in the MSD sector is as follows. A physiotherapist wishing to try out TR could use it when the patient no longer must come to the office to be treated, or when he/she is at the end of the rehabilitation process and should do some supervised exercises (hands-off). TR would, therefore, allow the physiotherapist to maintain contact with his/her patient while limiting the constraints of travel.

## Figures and Tables

**Table 1:** Descriptive data concerning the two groups (patients and physiotherapists). A * denotes a significant difference between both groups. Age is given under the form mean ± standard deviation. The last two lines are given under the form median [Q1–Q3].

Parameter	Patients	Physiotherapists	*p*
*n*	68	107	
Age (years)	46.6 ± 14.1	31.6 ± 10.0	≤0.001 *
Live or work in (Belgium–France)	56%–44%	78%–22%	0.004 *
Adequate Internet connection quality (Yes/No)	89%–11%	94%–6%	0.260
Adequate ease in using the Internet (Yes/No)	98%–2%	98%–2%	0.690
Previous use of TR (Yes/No)	88%–12%	76%–24%	0.065
Number of managed MSD patients per week		15 [10,11,12,13,14,15,16,17,18,19,20,21,22,23,24,25,26,27,28,29,30]	

## Data Availability

Data are available at https://osf.io/dn6qm/. Last accessed on 1 November 2021.

## Appendix A. List of Items

The participants were asked to give their opinion regarding the items listed in Table A1.

**Table A1:** Summary of the items assessing the perceived ease-of-use of TR (first line), its perceived usefulness (second to fifth lines), and participants’ behavioral intention (last line).

TAM3	Item
Perceived ease-of-use	I think it would be difficult for me to master any of the tools needed for TR.
Image	I would be willing to be managed/manage my patient by at least one means of MSD TR.
Job relevance	TR seems to me to be a favorable alternative for the management of MSD.
	I support TR only as a complement to face-to-face sessions.
Output quality	I support TR as a complete replacement for face-to-face sessions.
	I would be willing to pay/to charge the full authorized rate for the management of my MSD via TR.
Result demonstrability	TR makes the management of MSD less pleasant.
	I think my MSD/my patient’s MSD could be fully cured with TR sessions alone.
	I feel that TR would be beneficial to my treatment/my patient’s treatment.
	I believe that TR could improve the effectiveness of my treatment/my patient’s treatment.
Behavioral intention	I intend to use TR for the management of my MSD/my patient’s MSD.
